# Supplementary figures and images for: Robust and automatic definition of microbiome states
Source: PeerJ. 2019 Mar 26;7:e6657. doi: 10.7717/peerj.6657 (PMC6440462; doi:10.7717/peerj.6657)

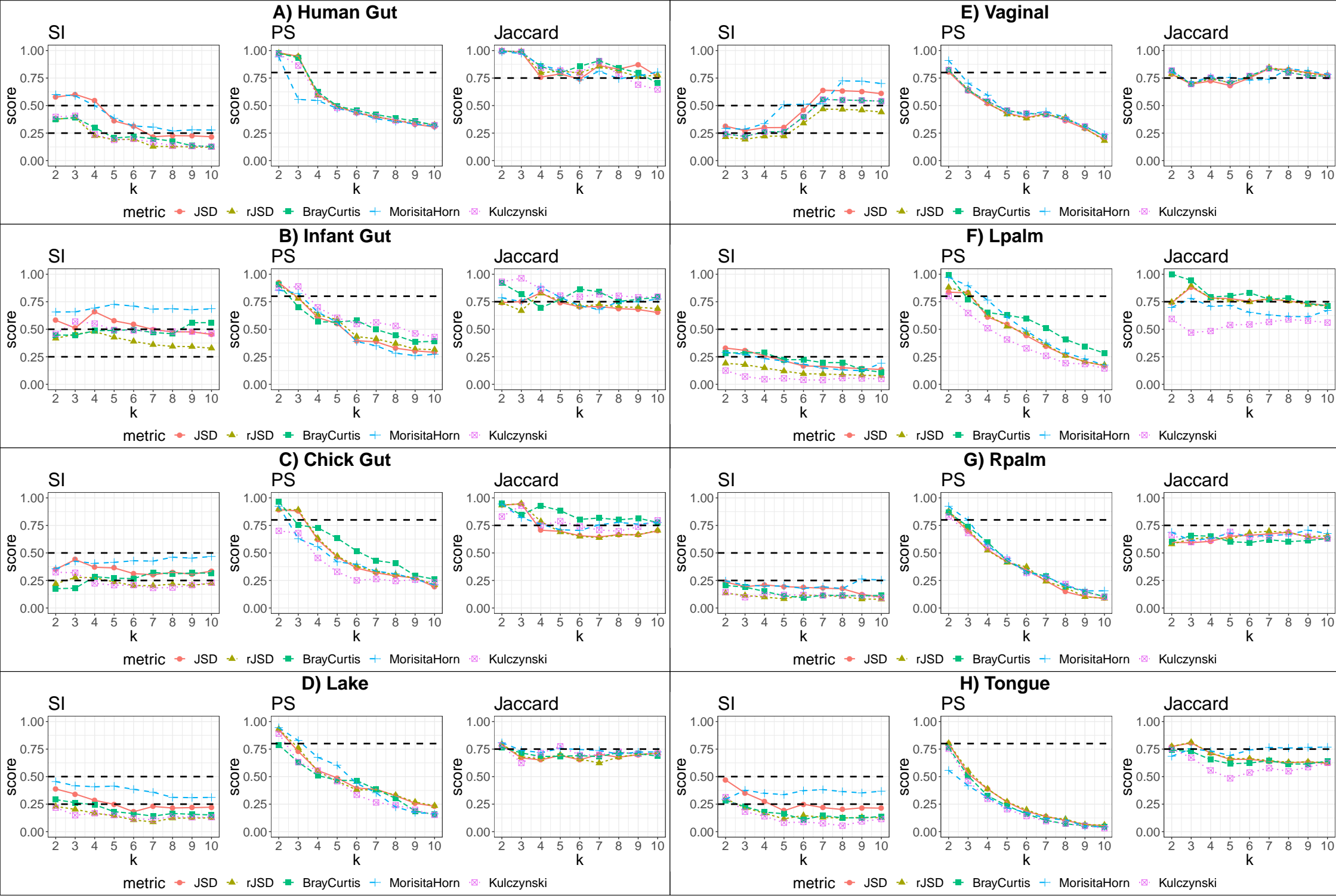

Supplement: Figure S1 [file peerj-07-6657-s001.pdf]

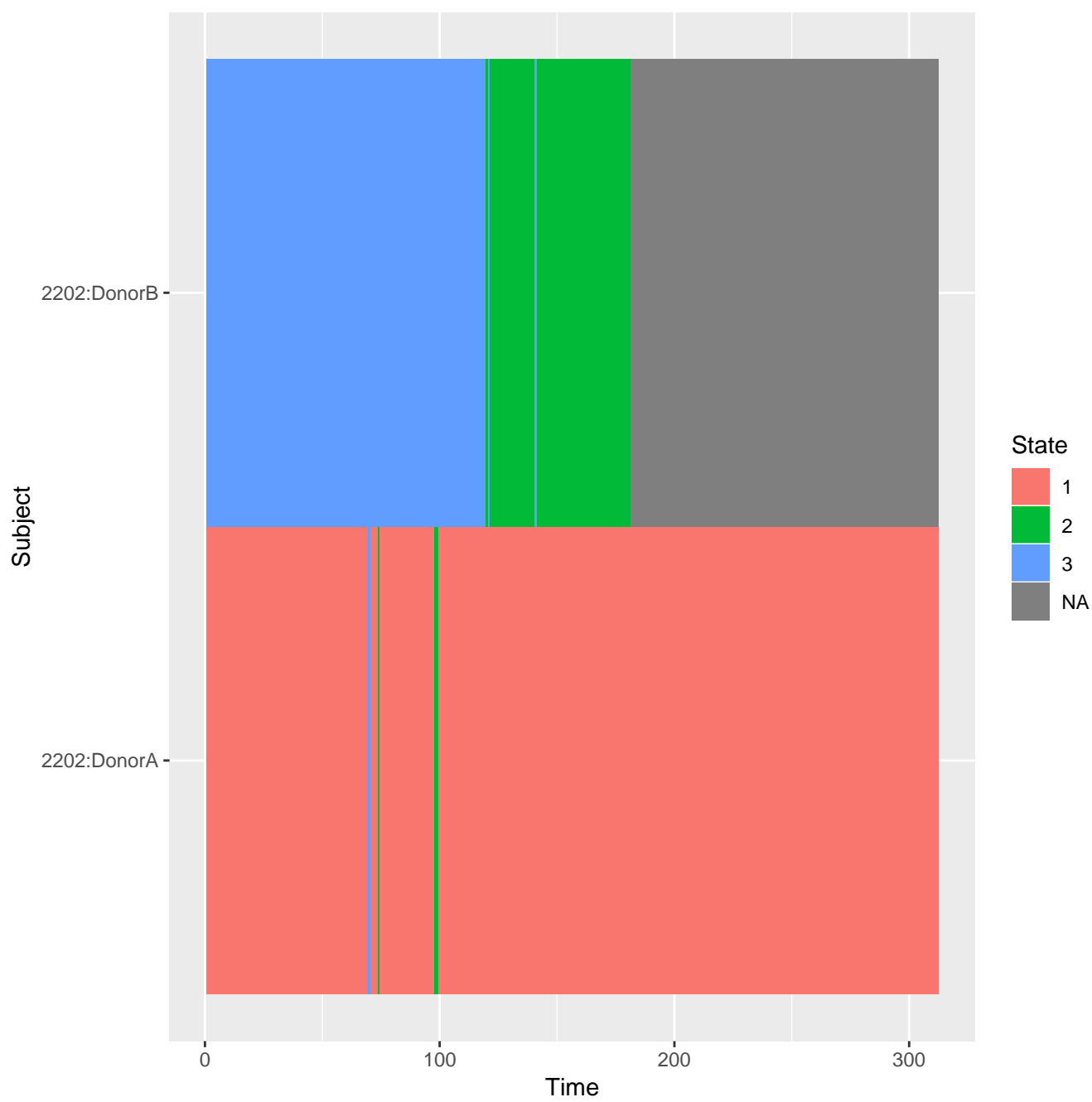

Supplement: Figure S2 — Samples sorted by time. Grey points represents no additional time point for that subject. [file peerj-07-6657-s002.pdf]

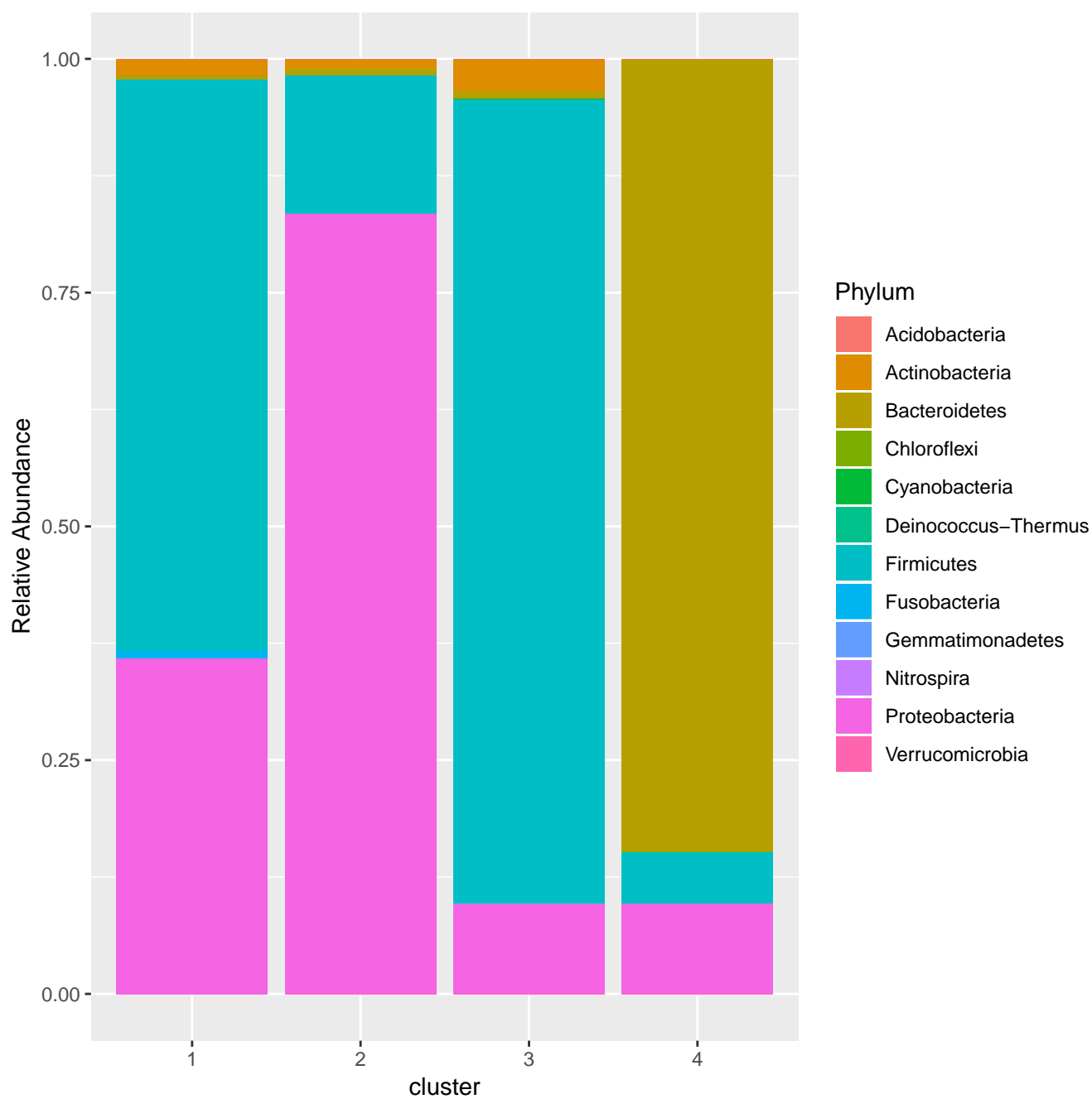

Supplement: Figure S3 [file peerj-07-6657-s003.pdf]

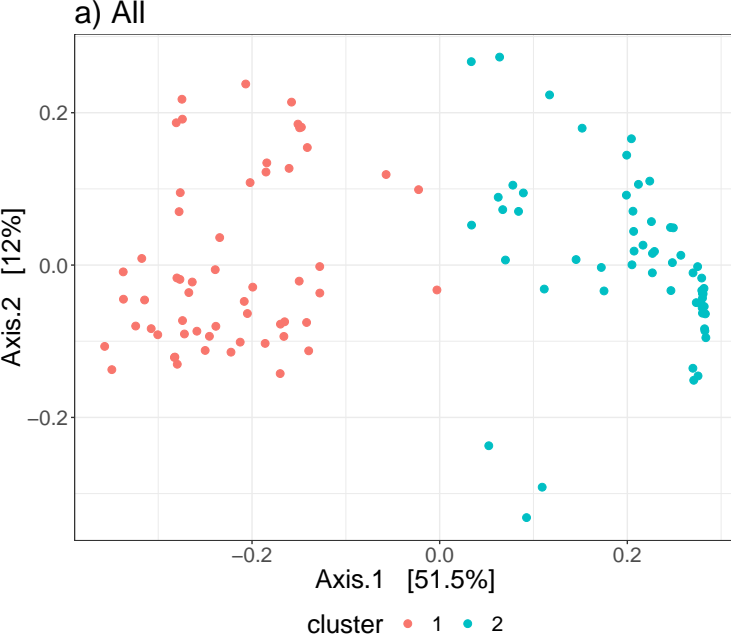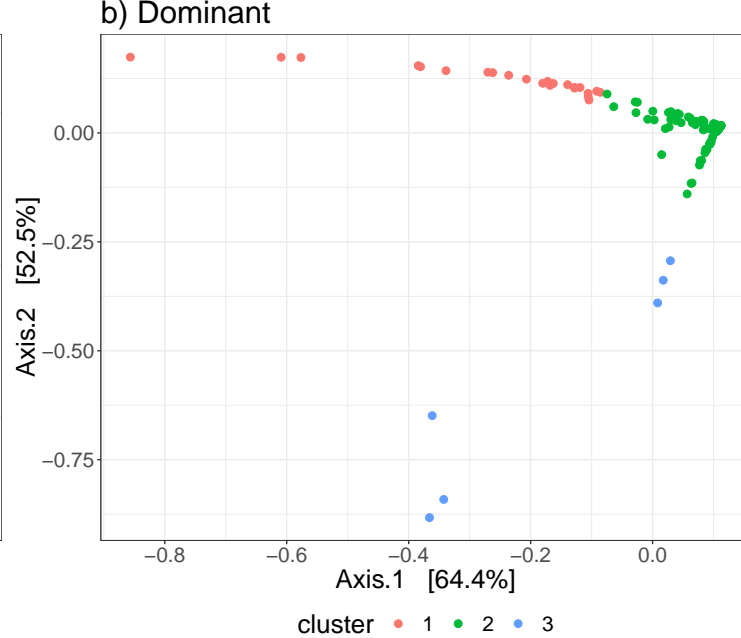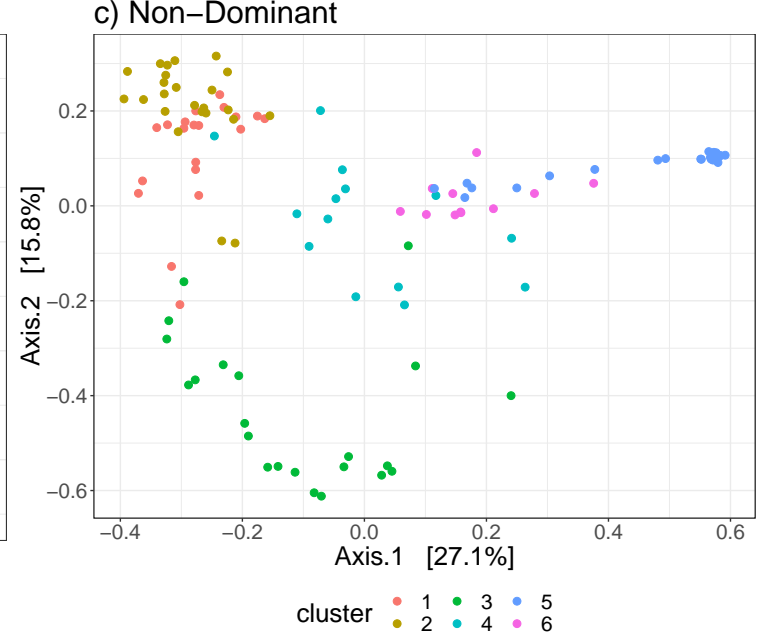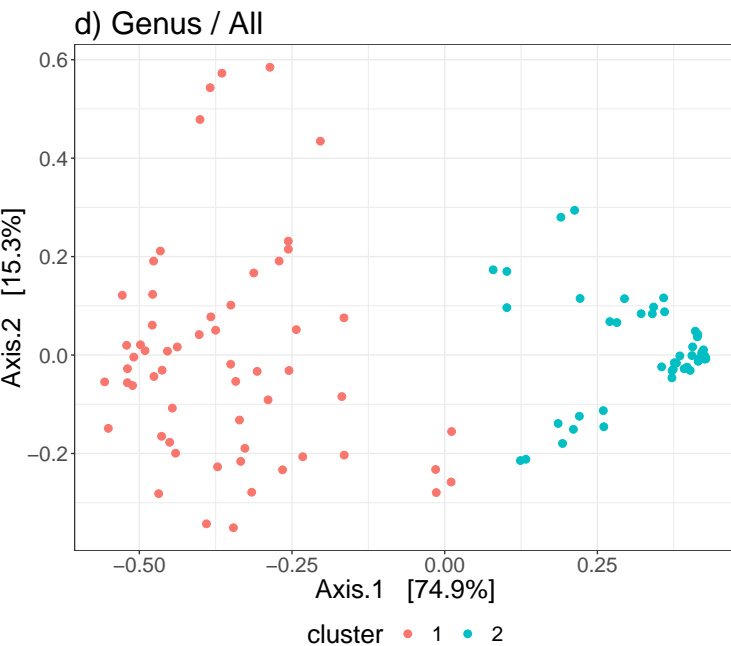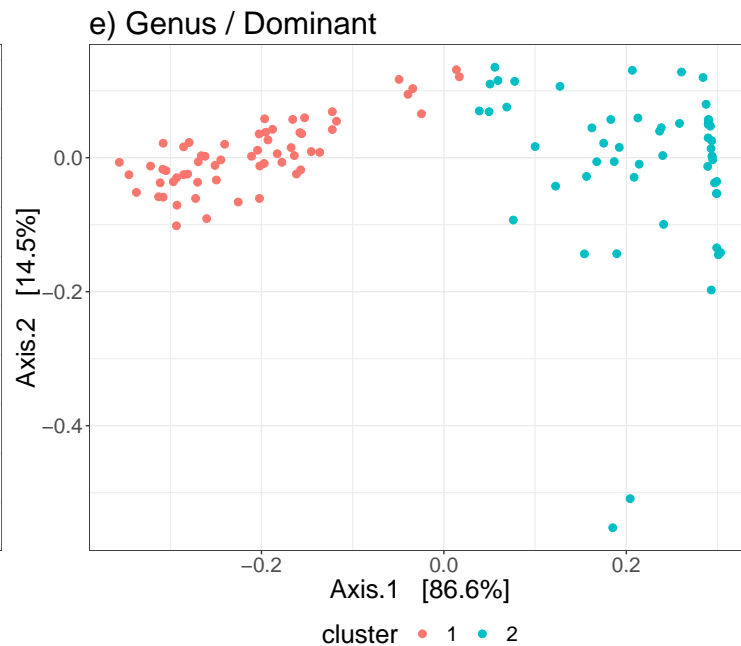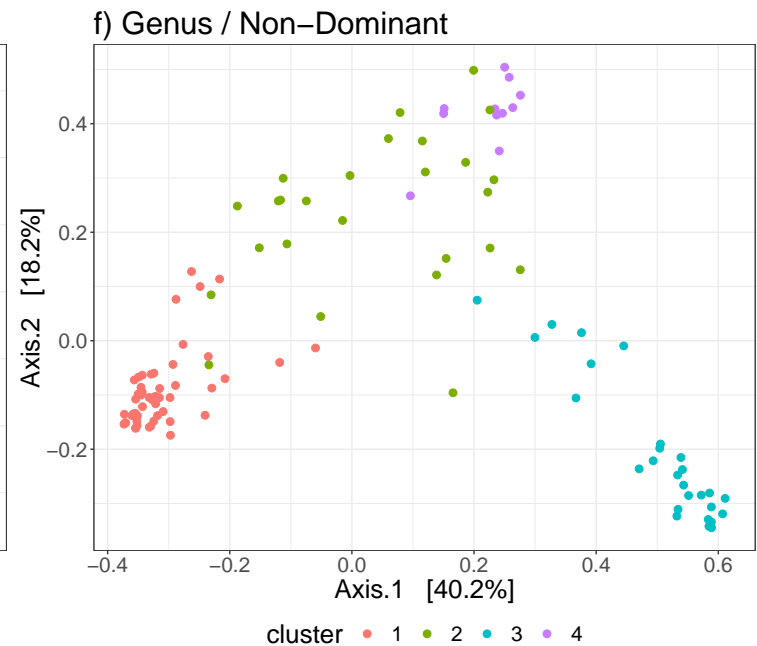

Supplement: Figure S4 — (A–C): Species level data. (D–F): Genus-level aggregation. [file peerj-07-6657-s004.pdf]

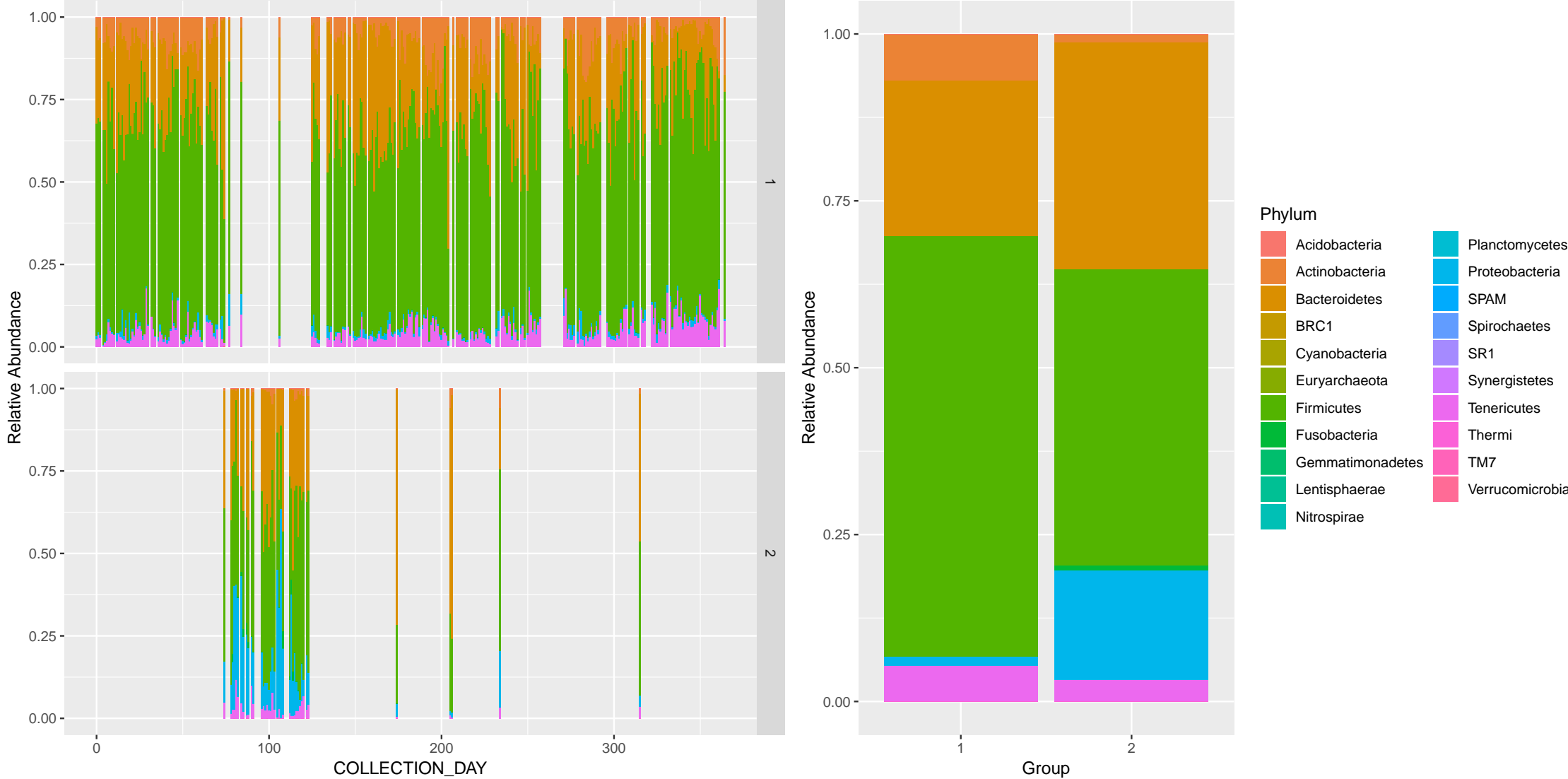

Supplement: Figure S5 — Samples sorted by collection day or grouped by clusters. [file peerj-07-6657-s005.pdf]

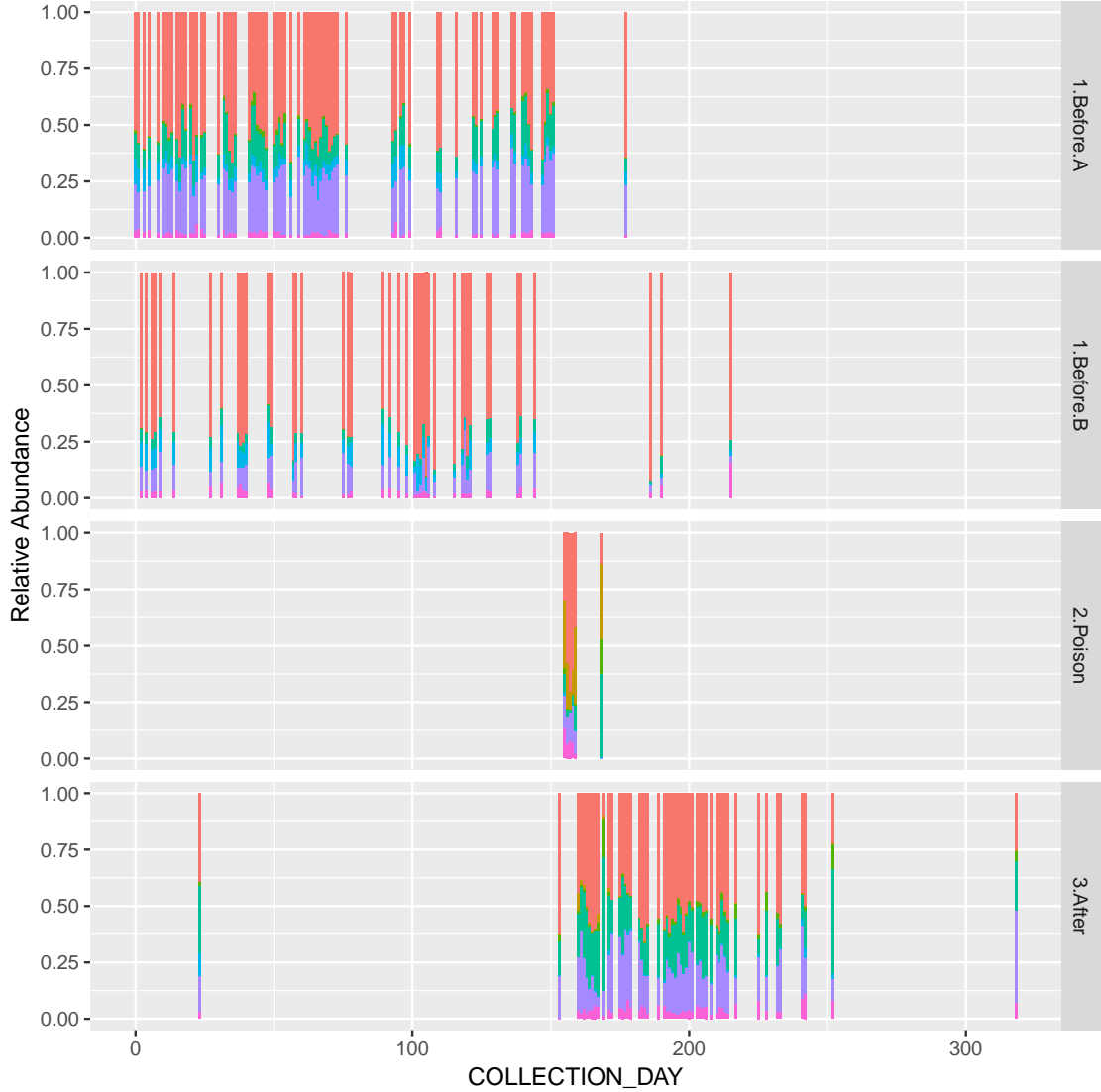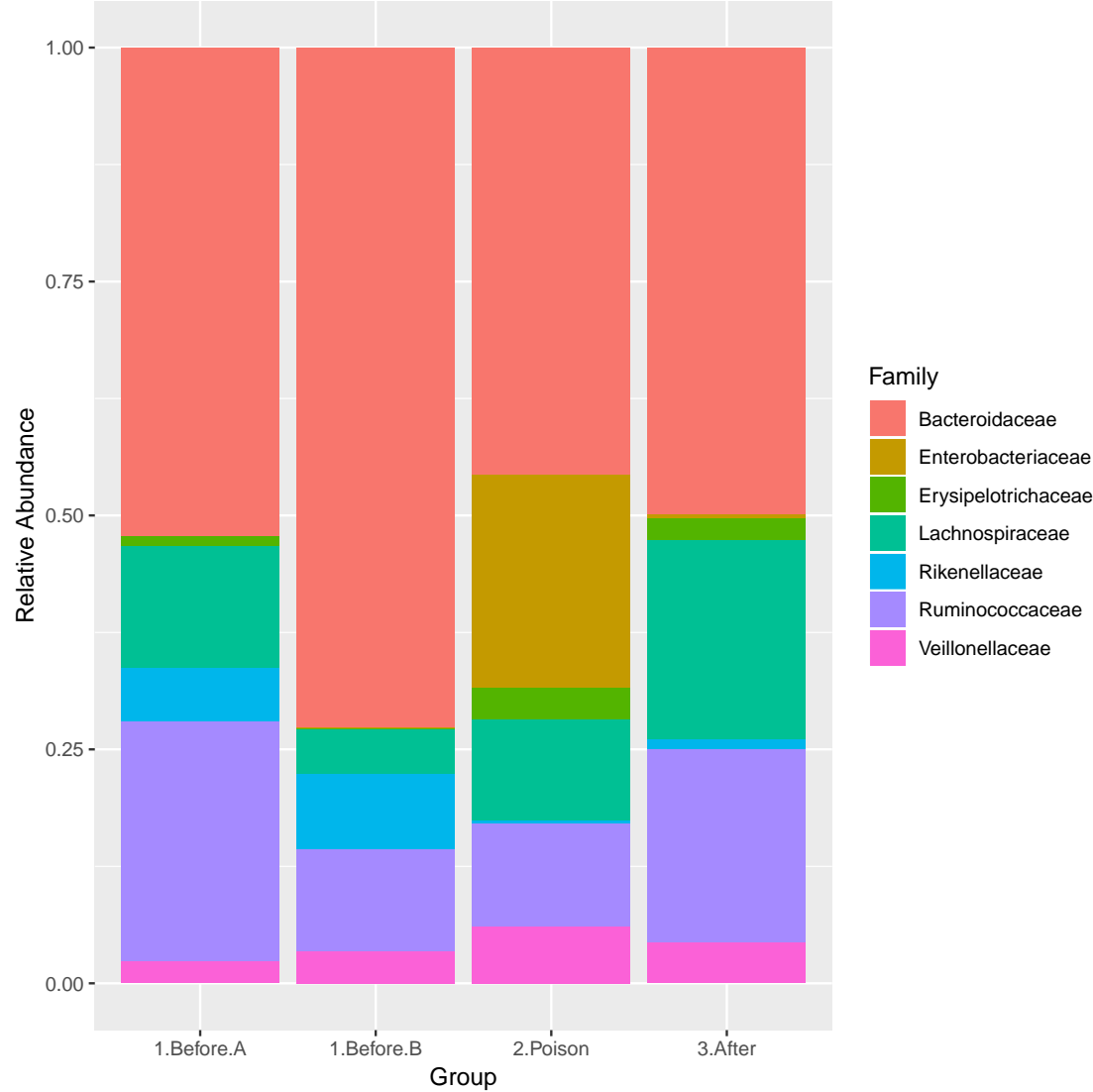

Supplement: Figure S6 — Samples sorted by collection day or grouped by clusters. [file peerj-07-6657-s006.pdf]
