# Supplementary material for: Robust and automatic definition of microbiome states
Source: PeerJ. 2019 Mar 26;7:e6657. doi: 10.7717/peerj.6657 (PMC6440462; doi:10.7717/peerj.6657)
Supplement: Table S1 [file peerj-07-6657-s007.pdf]

Table S1

Table S1: **Comparison between microbiome states defined in the current study vs those ones defined in (Gajer *et al.*, 2012).**

	**Current states**			
**Gajer2012**	**4**	**2**	**1**	**3**
**I**	162	0	0	1
**II**	0	57	0	0
**III**	4	0	360	15
**IV-A**	38	5	31	17
**IV-B**	0	0	0	247
